# Alteration in perceived health status of those aged 55 to 65 between 2010 and 2017 in France: role of socioeconomic determinants

**DOI:** 10.1186/s12889-021-11774-w

**Published:** 2021-10-07

**Authors:** Laure Carcaillon-Bentata, Noémie Soullier, Nathalie Beltzer, Joël Coste

**Affiliations:** Santé publique France, French national public health agency, F-94415 Saint-Maurice, France

**Keywords:** Self-reported health, Chronic condition, Limitations, Retirement, Social inequalities

## Abstract

**Background:**

While life expectancy increases, it is necessary to evaluate whether the additional years are lived in good health, particularly in order to adapt the health care provision and social measures available to support these individuals. Since the 1990s, improvements in perceived health and capacities have been observed among older people, however the changes appear to be far less favourable among the working-age population and, in particular, the generation of people approaching retirement age. The aim of this study was to examine the change in the perceived health status of those aged 55 to 65 in France and investigate the role of socioeconomic factors in this change.

**Methods:**

Self-reported health (SRH), chronic condition and activity limitation were assessed in 2010 and in 2017 in the French Health Barometer, a general population phone survey conducted on over 25,000 persons living in the community. The prevalence of all three indicators in 2017, and their evolution between 2010 and 2017 were studied according to the main socioeconomic determinants.

**Results:**

Between 2010 and 2017, there was a sharp increase in the proportion of individuals aged 55–65 reporting poor SRH, chronic condition or activity limitation. A much more marked deterioration was observed in the three indicators among those aged 55–65 than in older age groups, as well as different changes according to socioeconomic determinants. The category of workers with an average level of education was particularly affected by the deterioration.

**Conclusions:**

This study confirms that the generation approaching retirement age presents a more significant deterioration in health status than those of previous generations. The question of how these trends will be reflected in terms of the burden of dependency at later ages remains open and should encourage increased monitoring of and prevention among this population in future years.

**Supplementary Information:**

The online version contains supplementary material available at 10.1186/s12889-021-11774-w.

## Background

In the current context of a strong increase in the proportion of older people in the population due to the combined effect of the ageing of numerous baby boom generations and an increase in life expectancy, it is essential to be able to anticipate the burden of disability and, more generally, ill health, in order to adapt the health care provision and social measures available to support these individuals. Since the 1990s, disparate changes have been observed in chronic conditions, activity limitations and perceived health indicators in western countries, especially according to age group. As a result, despite the increase in the burden of chronic conditions due to population ageing, an improvement can be seen in disability levels among older people [1–3]. These trends reflect the improvement in the diagnosis and care of certain disabling illnesses and a reduction in mortality. However, very little research has been carried out on these trends among working-age populations and, in particular, the generation of people approaching retirement age. There have been very few studies on the change in health status and functioning in this population but they suggest that there has been no improvement, or even that there has been a deterioration in their health status in comparison with previous generations [1].

In France, similar trends were observed with a general stability in the evolution of disability-free life expectancy between 2004 and 2015 [4]. In addition, in line with other international studies, an expansion of disability in mid-adulthood (50–65 years) was reported for the period 2003–2008 [5]. To our knowledge, this worrying trend has not been reevaluated in more recent years in France. Consistent with those previous findings, one recent study found a general decline in health-related quality of life (not disability) between 1995 and 2016 especially in younger and in employed subjects [6].

The aim of this study was to assess, at a population level, the recent evolution in self-perceived health in individuals approaching retirement age (55–65 years). Due to the considerable importance of socioeconomic factors, which strongly influence access to health care, environmental exposure and health-related behaviour [7, 8], specific attention was paid to the differences in the indicators evolution according to socioeconomic determinants.

## Methods

### Data sources

The study relies on data from two independent editions of the Santé publique France Health Barometer surveys conducted in 2010 and 2017. Since 1992, French Health Barometers have been used to survey the French general population to provide a better understanding of health-related perceptions, attitude and behaviour, with a view to guiding public awareness and prevention policies. They therefore address numerous topics such as nutrition, smoking, high blood pressure, sleep and mood disturbance, living conditions, etc. They take the form of repeated cross-sectional telephone surveys.

The sampling frame is based on a random digit dialing of landline and mobile telephone numbers. To be part of the survey, individuals have to live in mainland France and speak French. In 2010, people aged 15 to 85 years were interviewed; in 2017, individuals had to be aged 18 to 75 years to be interviewed. Residents of collective dwellings, hospitals and institutions were excluded from the target population. In 2017, the participation rate was 48.5% and the Barometer included a sample of 25,319 individuals [9]. In 2010, the participation rate was 50.8% and the final sample comprised 27,653 individuals [10].

In both surveys, data were weighted to be representative of the French adult population. Data were adjusted by calibration to match the French population structure in terms of age, sex, region, town size, education level and number of persons per household, as based on the data of the 2008 and 2016 Labour force survey of the French population [11]. The detailed methodology of both surveys and questionnaires are available online [9, 10, 12, 13].

### Indicators of perceived health

Perceived health was measured according to the three measures included in the Minimum European Health Module (MEHM): self-reported health, reported chronic disease and global activity limitation [5]. The MEHM, which comprises three questions used to assess general health, the presence of chronic conditions and activity limitations, was developed to respond to the need for parsimonious and standardised indicators to facilitate the monitoring and comparison of changes in the different dimensions of health at national and international levels [14]. Nowadays, this method is increasingly used within the context of monitoring and assessing health policies [15]. The “perceived general health” question (or Self-reported health (SRH), How is your health in general?: Very good / Good / Fair / Bad / Very bad) is an assessment of a person’s own health. It is a global measure that has been used since the mid-1980s [16], incorporating the subject’s perceptions of physical, mental and social dimensions of health which have been associated with the occurrence of illnesses, with functional capacity, with mortality and with health care consumption [16–20]. The reporting of a chronic condition (Do you have any chronic or long-standing illnesses or health problems?: Yes/No) is obviously determined by the knowledge and diagnosis of illnesses. Beyond this knowledge, this reliable indicator [21, 22] reflects the way people feel about their problems and their health care needs. Lastly, the third question relates to activity limitations (or the Global Activity Limitation Indicator, GALI: For at least the past 6 months, have you been limited in activities people usually do because of a health problem?: Yes, severely limited / Yes, limited, but not severely / No, not limited at all). This question, equally predictive of mortality and health care expenditure [23] is probably the one that has been most commonly used to date, especially in the context of calculating disability-free life expectancy in Europe. These three measures, especially the two indicators [24] for general health and activity limitations have been used to identify and characterise health inequalities in Europe [25, 26].

In the 2017 questionnaire, the three questions related to SRH, chronic disease and limitation were grouped within a same module; in the 2010 survey, the three questions were distributed throughout the questionnaire (supplementary file 1). Additionally, in 2010, the question regarding SRH was asked to a subsample of 9110 individuals. Indeed, as a multi-thematic repeated survey, the French health Barometer is divided into random subsamples in order to be able to address many themes within the same survey by reducing the length of the questionnaire and the cost of the survey. Calibrated weights are calculated for each random samples in order to perform adequate statistical analyses.

Regarding SRH, in line with the WHO recommendations [27] and those of the ECHIM project, [28] response were separated in two categories, corresponding to ‘good’ SRH, and ‘poor’ SRH (including all categories of responses less than “good”). With regard to activity limitations, the responses “yes, a little” and “yes, severely” were separated from the response “no, not at all”.

### Statistical analysis

Descriptions of the 2010 and 2017 full samples of the barometer surveys are available elsewhere [9, 10]. Individual retirement age was not available in the survey data. As the objective of the study was to investigate changes in people’s perceived health at the time of retirement, the present analyses primarily focused on those aged 55 to 65. In this age-group, subjects are close to their retirement age which has evolved from 60 to 62 in 2010. Overall, 5429 individuals surveyed in 2010 and 5802 in 2017 were included in the present study. In 2010, the question regarding SRH was posed to a subsample of 1795 individuals aged 55 to 65. A full description of all 3 samples is provided in supplementary file 2.

First, the perceived health status of those aged 55 to 65 in 2017 was described using frequencies and prevalences for the three indicators (SRH, chronic condition and activity limitations) among all those aged 55 to 65, then by subpopulation, defined by the following variables: sex (male/female), living alone (yes/no), marital status (married or in a civil partnership/single/divorced/widowed), education level (below the baccalaureate/baccalaureate or equivalent/above the baccalaureate), employment status (employed/retired/unemployed/non-working), socioprofessional category (current occupation or last occupation in 5 categories), professional status (employed/self-employed), income per unit of consumption (in tertiles) and perceived financial status (difficult/adequate/satisfactory). The confidence intervals were calculated according to the Clopper-Pearson method. The prevalences in the different subpopulations were compared using a weighted chi-square test.

Second, in order to identify independent associations between socio-economic variables and all three indicators, multivariate weighted Poisson regressions including age as well as all previously mentioned variables were performed using backward selection providing prevalence ratio (PR) and their 95% confidence interval. The adjustment for age was linear; this adjustment proved satisfactory among the population of 55 to 65-year-olds. In order to study SRH, an additional model was developed, including chronic condition and activity limitation as adjustment variables as the presence of activity limitations or chronic diseases may explain a part of SRH.

Third, the change in perceived health status between 2010 and 2017 among those aged 55–65 was compared to those of other age groups. For this purpose, a weighted Poisson regression was performed for each indicator (self-rated health/chronic condition/activity limitation) adjusting for age, survey year and the interaction between age and survey year among those aged 18 to 75. In these analyses, age was taken into account using a second-degree fractional polynomial form and prevalence.

Fourth, the change between 2010 and 2017 in the perceived health status of those aged 55–65 was compared according to the above-mentioned socioeconomic characteristics. Bi-variate analyses were performed for each variable of interest (self-rated health/chronic condition/activity limitation) by means of weighted Poisson regressions, adjusted for age (linear), the survey year, the socioeconomic characteristic being studied, and the interaction term between the survey year and the socioeconomic variable when the specific effects of these two variables were significant. Multivariate analyses for each variable of interest was further performed using a backward selection. The interaction term on the multiplicative scale, raised to the exponential, corresponding to a relative risk ratio (RRR) [29],represents the parameter of interest for studying the differences in the changes between the groups studied. Again, adjustment for activity limitation and chronic disease was made when studying SRH. These analyses were stratified by sex.

The analyses take into consideration the adjusted weightings for the years 2010 and 2017. Variance estimations in Poisson regression models are performed using the robust method [30, 31].

The analyses were performed using the software SAS 9.4 (for prevalence analysis) and Stata 14.2 (for modelling). The graphics were created using R 3.4.3. software.

## Results

In 2017, half of those aged 55–65 reported that they had a chronic condition, 35% reported poor SRH and 29% felt that they were limited in activities people usually do (Table 1).

**Table 1:** Weighted prevalence of each indicator of the Minimum European Health Module among those aged 55–65 in 2017 according to socioeconomic determinants

	Poor Self-reported Health	Chronic condition	Activity limitaiton
All those aged 55–65 (*n* = 5802)	34.7 [33.2; 36.3]	49.7 [48.7; 51.9]	28.9 [27.4; 30.4]
**Sex**	***		***
Male (*n* = 2568)	32.0 [29.7; 34.3]	49.0 [46.6; 51.4]	26.7 [24.6; 28.8]
Female (*n* = 3234)	37.3 [35.2; 39.5]	50.4 [48.2; 52.5]	31.0 [28.9; 33.1]
**Lives alone**	***	**	***
Does not live alone (*n* = 4241)	32.7 [30.9; 34.5]	48.8 [46.9; 50.6]	27.4 [25.7; 29.1]
Lives alone (*n* = 1561)	42.6 [39.4; 45.8]	53.3 [50.1; 56.5]	34.7 [31.7; 37.8]
**Marital status**	***	*	***
Married or in a civil partnership (*n* = 3521)	30.9 [29.0; 32.8]	48.1 [46.1; 50.1]	26.2 [24.4; 28.0]
Single (*n* = 1016)	38.0 [33.8; 42.3]	51.5 [47.2; 55.8]	32.8 [28.7; 37.1]
Divorced (*n* = 913)	43.9 [39.8; 48.1]	52.9 [48.7; 57.0]	33.9 [30.0; 37.9]
Widowed (*n* = 346)	47.1 [40.0; 54.3]	54.9 [47.9; 61.8]	35.6 [29.0; 42.5]
**Education level**	***	***	***
Below the baccalaureate (*n* = 2905)	40.4 [38.2; 42.6]	51.6 [49.4; 53.9]	31.9 [29.9; 34.0]
Baccalaureate or equivalent (*n* = 1015)	27.9 [24.7; 31.2]	46.6 [43.0; 50.1]	25.4 [22.4; 28.6]
Above the baccalaureate (*n* = 1870)	22.8 [20.6; 25.2]	46.2 [43.5; 48.9]	22.3 [20.1; 24.6]
**Employment status**	***	***	***
Employed (*n* = 2756)	26.4 [24.4; 28.6]	43.6 [41.3; 46.0]	21.5 [19.6; 23.4]
Unemployed (*n* = 317)	45.3 [38.5; 52.2]	48.8 [42.0; 55.7]	35.0 [28.7; 41.7]
Retired (*n* = 2182)	33.6 [31.1; 36.1]	49.9 [47.3; 52.5]	26.3 [24.1; 28.6]
Non-working (*n* = 547)	65.5 [60.4; 70.3]	73.6 [68.7; 78.1]	63.4 [58.0; 68.5]
**Socioprofessional category**	***	**	***
Farmer, artisan, merchant, entrepreneur (*n* = 506)	31.0 [26.2; 36.1]	45.0 [39.7; 50.4]	20.6 [16.6; 25.0]
Executive, senior intellectual profession (*n* = 1023)	22.4 [19.5; 25.6]	47.0 [43.4; 50.6]	21.6 [18.7; 24.7]
Intermediary profession (*n* = 1586)	28.5 [25.8; 31.4]	47.7 [44.7; 50.7]	27.0 [24.5; 29.7]
Employee (*n* = 1636)	38.6 [35.6; 41.6]	49.9 [46.9; 52.9]	29.8 [27.0; 32.6]
Labourer (*n* = 1024)	43.4 [39.7; 47.2]	54.1 [50.3; 57.8]	35.8 [32.2; 39.5]
**Status**	***		***
Employed (*n* = 5218)	34.9 [33.3; 36.6]	49.4 [47.7; 51.1]	29.3 [27.8; 30.9]
Self-employed (*n* = 527)	27.7 [23.2; 32.6]	48.2 [42.9; 53.6]	20.7 [16.7; 25.1]
**Revenue per unit of consumption in tertiles**	***	***	***
1st tertile (low)(*n* = 1351)	48.4 [45.0; 51.8]	54.8 [51.3; 58.2]	38.6 [35.4; 42.0]
2nd tertile (*n* = 1794)	34.7 [32.1; 37.4]	49.9 [47.1; 52.6]	27.2 [24.8; 29.7]
3rd tertile (high) (*n* = 2419)	22.6 [20.6; 24.7]	45.9 [43.5; 48.3]	21.8 [19.9; 23.9]
Don’t know/refusal (*n* = 238)	35.9 [28.4; 43.9]	44.4 [36.4; 52.7]	28.9 [22.0; 36.6]
**Perceived financial status**	***	***	***
Difficult (*n* = 633)	57.1 [51.9; 62.1]	63.0 [57.8; 67.9]	42.8 [37.7; 48.0]
Adequate (*n* = 1195)	41.6 [38.2; 45.1]	54.5 [51.1; 58.0]	35.6 [32.3; 38.9]
Satisfactory (*n* = 3951)	27.6 [25.9; 29.4]	45.2 [43.3; 47.1]	23.6 [22.0; 25.2]
**Chronic condition**	***		
No (*n* = 2961)	16.0 [14.3; 17.8]		
Yes (*n* = 2826)	53.5 [51.2; 55.7]		
**Activity limitation**	***		
No (*n* = 4181)	21.6 [20.0; 23.3]		
Yes (*n* = 1614)	67.2 [64.3; 70.0]		

*Significance level < 0.1, **Significance level < 0.05, ***Significance level < 0.01 – Weighted chi-square test

### Analysis of perceived health determinants in 2017

Women aged 55–65 reported poor SRH and activity limitations more frequently than men of the same age. Living alone, not having any diploma or a level of education below baccalaureate, being non-working, a labourer, having a low income or financial difficulties was significantly associated with poorer perceived health among those aged 55–65, for the three indicators considered. These associations remained after adjustment for sex and age. Being widowed or divorced, unemployed or retired, and being employed rather than self-employed, was more commonly associated with poor SRH and activity limitation. Lastly, executives and those in intermediary professions less often reported poor SRH; chronic condition and activity limitation were reported less frequently among artisans and farmers. These associations remained after adjustment for sex and age (Table 1).

The multivariate model results are presented in Table 2. Only education level, employment status, income level or financial difficulties were independently associated with perceived health status. An education level below the baccalaureate (PR = 1.29 [1.15; 1.43]), being unemployed (PR = 1.22 [1.04; 1.43]) or non-working (PR = 1.22 [1.10; 1.36]), having an income in the first and second tertiles (PR = 1.33 [1.17; 1.51] and PR = 1.24 [1.11; 1.39], respectively) and having financial difficulties (PR = 1.24 [1.11; 1.38]) were independently associated with poor SRH, after adjustment for reported chronic disease and activity limitation. Only employment status and the level of financial difficulties were significantly associated with reporting a chronic condition: being retired (PR = 1.11 [1.02; 1.22]) or non-working (PR = 1.58 [1.46; 1.72]), reporting an adequate financial status (PR = 1.17 [1.09; 1.26]) or having financial difficulties (PR = 1.31 [1.20; 1.43]) were independently associated with a higher risk of reporting a chronic condition. Lastly, employment status and socioprofessional category were the only two variables independently associated with reporting activity limitations: being unemployed (PR = 1.40 [1.15; 1.72]), retired (PR = 1.18 [1.02; 1.37]) or non-working (PR = 2.66 [2.36; 3.02]) and being in an intermediary profession (PR = 1.23 [1.04; 1.45]), being an employee (PR = 1.26 [1.07; 1.48]) or a labourer (PR = 1.33 [1.12; 1.58]) were independently associated with a higher risk of presenting activity limitations, when compared to employed and executive, respectively.

**Table 2:** Prevalence ratio (PR) for the Minimum European Health Module indicators among those aged 55–65 in 2017 based on socioeconomic determinants

	Poor Self-reported Health		Chronic condition Model 1 PR [95% CI]	Activity limitation Model 1 PR [95% CI]
	Model 1 PR [95% CI]	Model 2 PR [95% CI]		
**Sex** (Male/Female)	NS	NS	NS	NS
**Lives alone** (Yes/No)	NS	NS	NS	NS
**Marital status** (Married/Single/Divorced/Widowed)	NS	NS	NS	NS
**Education level**	***	***	NS	NS
Below the baccalaureate	1.31 [1.16; 1.48]	1.29 [1.15; 1.43]		
Baccalaureate or equivalent	1.08 [0.93; 1.26]	1.09 [0.95; 1.24]		
Above the baccalaureate (ref)	–	–		
**Employment status**	***	***	***	***
Employed (ref)	–	–	–	–
Unemployed	1.34 [1.13; 1.59]	1.22 [1.04; 1.43]	1.04 [0.90; 1.21]	1.40 [1.15; 1.72]
Retired	1.15 [1.01; 1.31]	1.05 [0.93; 1.18]	1.11 [1.02; 1.22]	1.18 [1.02; 1.37]
Non-working	1.96 [1.74; 2.19]	1.22 [1.10; 1.36]	1.58 [1.46; 1.72]	2.66 [2.36; 3.01]
**Socioprofessional category**	NS	NS	NS	***
Farmer, artisan, merchant, entrepreneur				0.92 [0.72; 1.17]
Executive, senior intellectual profession (ref)				–
Intermediary profession				1.23 [1.04; 1.45]
Employee				1.26 [1.07; 1.48]
Labourer				1.33 [1.12; 1.58]
**Status** (Employed/Self-employed)	NS	NS	NS	NS
**Revenu per unit of consumption in tertiles**	***	***	NS	NS
1st tertile (low)	1.36 [1.18; 1.56]	1.33 [1.17; 1.51]		
2nd tertile	1.26 [1.11; 1.42]	1.24 [1.11; 1.39]		
3rd tertile (high) (ref)	–	–		
Don’t know/refusal	1.25 [0.99; 1.57]	1.35 [1.11; 1.64]		
**Perceived financial status**	***	***	***	***
Difficult	1.53 [1.35; 1.72]	1.24 [1.11; 1.38]	1.31 [1.20; 1.43]	1.42 [1.24; 1.62]
Adequate	1.27 [1.14; 1.41]	1.09 [0.99; 1.20]	1.17 [1.09; 1.26]	1.35 [1.21; 1.51]
Satisfactory (ref)	–	–	–	–
**Chronic condition**	Not included	***	Not included	Not included
No (ref)		–		
Yes		2.37 [2.10; 2.67]		
**Activity limitation**	Not included	***	Not included	Not included
No (ref)		–		
Yes		2.01 [1.83; 2.21]		

***Significance level < 0.01

Model 1: Poisson model with linear adjustment for age and significant explanatory variables (*p*-value < 0.05)

Model 2: Model 1 with adjustments for “chronic condition” and “activity limitation” variables

Abbreviations: Confidence Interval, CI; Not significant, NS

Sex, living alone, marital status and professional status (employed/self-employed) were not independently associated with either poor SRH or reporting chronic condition or activity limitation. These variables were not used in the comparison of changes in health status between 2010 and 2017 based on socioeconomic and demographical characteristics.

### Changes in health status between 2010 and 2017 according to age among those aged 18–75

**Fig. 1:**
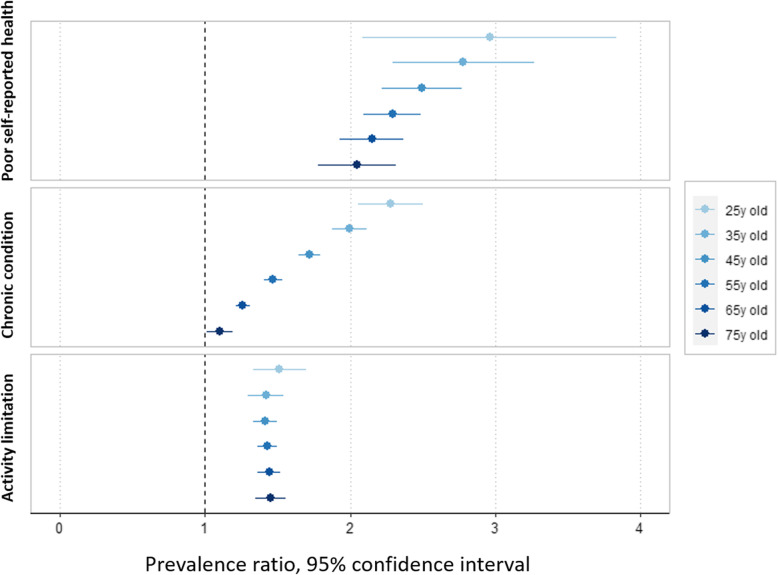
Prevalence ratios between 2010 and 2017 for the three Minimum European Health Module indicators based on age

Between 2010 and 2017, there was a sharp increase in the proportion of individuals aged 55–65 reporting poor SRH, chronic condition or activity limitation (PR = 2.24 [1.93; 2.59], PR = 1.38 [1.31; 1.45] and PR = 1.42 [1.31; 1.53], respectively. Supplementary file 2). Figure 1 shows a deterioration in health status between 2010 and 2017 for all ages. The deterioration was significantly higher among younger people for the presence of a chronic condition (*p*-value of age x year interaction < 0.001) and for poor SRH (*p*-value = 0.06). The increase in the reporting of limitations was lower and identical for all age groups.

The same trends were identified among men and women; only the deterioration in the presence of chronic condition was slightly higher among younger women than younger men (*p*-value< 0.0001).

### Changes in health status according to socioeconomic characteristics between 2010 and 2017 among those aged 55–65

Results regarding bi-variate analyses are available in supplementary file 3. In multivariate analyses, the change in perceived health indicators varied between 2010 and 2017 based on education level, socioprofessional category, and employment status (Table 3). Overall, the relative risk for poor SRH increased by 85% among individuals with an education level equal to the baccalaureate compared to those with a higher education level (RRR = 1.85 [1.10; 3.10]), and by 31 and 44% among those with an occupation compared to those who were non-working or unemployed, respectively. On the contrary, the relative risk for activity limitation increased by 38% among non-workers compared to workers. The risk of poor SRH among workers was more pronounced among men and the risk of activity limitation among non-workers was more pronounced among women. Lastly, among men, the relative risk of activity limitation increased by 47% among those in intermediary professions and by 76% among labourers, compared to executives.

**Table 3:** Change in perceived health between 2010 and 2017 according to socioeconomic determinants (relative risk ratios and 95% confidence interval), multivariate models, among those aged 55–65, by sex

	Poor Self-reported health						Chronic Condition						Activity limitation					
	All		Men		Women		All		Men		Women		All		Men		Women	
	RRR	[95% CI]	RRR	[95% CI]	RRR	[95% CI]	RRR	[95% CI]	RRR	[95% CI]	RRR	[95% CI]	RRR	[95% CI]	RRR	[95% CI]	RRR	[95% CI]
**Education level (ref = > bac)**	***		***		***		**NS**		**NS**		**NS**		**NS**		**NS**		**NS**	
Education level * year	**		**NS**		**		**NA**		**NA**		**NA**		**NA**		**NA**		**NA**	
Below the bac * year	0.91	[0.68;1.21]			0.96	[0.67;1.38]												
Bac * year	**1.85**	[1.10;3.10]**			**2.09**	[1.07;4.06]**												
**Employment status (ref = employed)**	***		***		**		***		***		***		***		***		***	
Employment status * year	**		***				**NS**		**		**NS**		**		**NS**		**	
Unemployed * year	0.56	[0.32;1.01]	**0.37**	[0.20;0.70]***					**0.64**	[0.46;0.90]***			1.12	[0.79;1.58]			1.00	[0.64;1.52]
Retired * year	1.02	[0.75;1.40]	1.23	[0.78;1.95]					0.91	[0.76;1.09]			1.02	[0.85;1.23]			1.19	(0.52;1.52)
Non-working * year	**0.69**	[0.60;0.97]**	0.66	[0.38;1.12]					**0.82**	[0.66;1.30]*			**1.38**	[1.11;1.71]***			**1.53**	[1.16;1.99]***
**Socioprofessional categories (ref = executive)**	**NS**		**NS**		***		**NS**		**NS**		**NS**		*		**NS**		**NS**	
Socioprofessional categories * year	**NA**		**NA**		***		**NA**		**NA**		**NA**		**NS**		**		**NA**	
Farmer, artisan, merchant * year															1.35	[0.84;2.16]		
Intermediary profession * year															**1.47**	[1.04;2.08]**		
Employee * year															1.23	[0.80;1.87]		
Labourer * year															**1.76**	[1.24;2.48]***		
**Income**	***		**NS**		***		**NS**		**NS**		**NS**		**NS**		**NS**		**NS**	
Income * year	**NS**		**NA**		**NS**		**NA**		**NA**		**NA**		**NA**		**NA**		**NA**	
**Financial status (ref = satisfactory)**	***		***		***		***		***		***		***		***		***	
Financial status * year	**NS**		**NS**		**NS**		**NS**		**NS**		**NS**		**NS**		**NS**		**NS**	
Difficult * year																		
Adequate * year																		

*p-value < 0.1, **p-value < 0.05, ***p-value < 0.01

Abbreviations: Relative risk ratio, RRR; Confidence Interval, CI; Not significant, NS; Interaction not included in the model, as the specific effect of the determinant was not significant in the model without interaction, NA

## Discussion

### Main results

This study provides an in-depth analysis of the temporal changes, between 2010 and 2017, of the three indicators of perceived health included in the MEHM, in relation to socioeconomic factors, among those aged 55–65, approaching retirement age. This study is the first of this kind in France. For the 55–65 age group, the study shows a clear deterioration in the indicators, much more pronounced than in older age groups and, in particular, different changes according to socioeconomic determinants. It points out one category especially affected by the deterioration: workers with an average level of education.

### Socioeconomic determinants of perceived health

First of all, the study confirms the importance of socioeconomic determinants of perceived health in France. Already highlighted using multidimensional instruments for quality of life linked to health such as the SF-36 [32] or Promis-29 [33], disparities across education levels, employment status and income level were observed again here [34] with the indicators of the MEHM. Among those aged 55–65, perceived health appeared to be poorer particularly among non-workers and the unemployed, and in the employed, among labourers, farmers and artisans. These results reflect, for some, the well-known “healthy worker” selection effects which mean that people who are in better health remain in employment [35, 36] and the inverse effect for those in “independent” occupations [37]. Although perceived financial status and employment status are independently associated with the three indicators, income level and socioprofessional category appear to be primarily associated with SRH and activity limitations, respectively. These results highlight the different valence of the MEHM indicators, as well as the benefit of multiple and multidimensional indicators for assessing health inequalities in terms of perceived health and responding, at least in part, to criticism regarding the exclusive use of the SRH indicator [38].

### Deterioration in perceived health between 2010 and 2017 and age

Perceived health status deteriorated between 2010 and 2017 for the 3 indicators studied. This deterioration is observed regardless of sex and age but becomes more important as age decreases for the reporting of chronic condition and poor SRH. Consequently, those aged 55–65 experience a more significant deterioration in their perceived health status than older age groups. This difference in change is not observed for the “activity limitation” indicator. The general deterioration in perceived health in recent years, as well as the difference in change according to age group, had already been observed in France and in several other northern European countries [6]. Several American studies had also highlighted these trends over the course of the two previous decades [39]. The positive effect of retirement on the different dimensions of health was also demonstrated [40]. People in older age groups could be better informed and more inclined to report and seek care for health problems and disabilities than before, as is also shown by the decrease in disability-free life expectancy among those aged 50–65 in contrast with older people [5]. These differences in the evolution of perceived health with age were observed in both sexes, contrary to what has been reported in other studies [6].

### Deterioration in perceived health between 2010 and 2017 and socioeconomic factors

The different evolutions in perceived health between 2010 and 2017 according to several socioeconomic determinants observed in this study are particularly significant. Although labourers and those in intermediary professions reported a more significant deterioration in activity limitations, it was workers in general, and specifically those with an average level of education (baccalaureate level), who reported the most significant deteriorations in SRH. The observation of more unfavourable trends among those in employment may seem to contradict studies that demonstrate an association between unemployment and deterioration in health status, particularly through the onset of depression [41] or cognitive decline [42]. However, this more significant deterioration in perceived health status among those in employment (who nevertheless still have a better level of health than unemployed or non-working individuals) over recent years appears to be genuine and has already been observed in France [6] and in several European countries in the years following the 2008 economic crisis [43]. Several factors may be put forward to explain this observed change: on the one hand, the new vulnerability among those who retained their jobs due to an increase in feelings of insecurity, psychosocial risks and mental distress [44]; on the other hand, the reduction in stigma associated with lack of employment during the crisis, and the social protection system’s efficiency to limit the negative impact of unemployment on perceived health. However, the possibility that people belonging to less vulnerable groups, such as employed individuals, have a higher level of expectations regarding their health than more disadvantaged groups cannot be ruled out. Nevertheless, although it has been demonstrated that a single life or health event can have a more significant impact on the perceived health of individuals with a higher socioeconomic status, this difference in expectations cannot explain such large and significant variations between groups [38].

### Limitations of the study

This study has a number of limitations. First and foremost, time frame (7 years) is relatively short in this study in view of the temporal changes which may have been initiated during the 2008 crisis, or even earlier [6, 45]. Secondly, the availability of only two time points, which penalize the examination of secular trends [46]. Thirdly, a possible lack of power in the analysis of the less frequent factors. Lastly, several differences in the wording of questions and responses recorded between 2010 and 2017 (Supplementary file 1) have to be considered. With regard to chronic condition, the question headings were different, as was the placement of the question in the questionnaire. Obviously, these differences cannot explain the differences observed between subgroups, under the (reasonable) hypothesis that their impact was similar among the different subgroups compared.

## Conclusion

The results of this study, in line with the currently available data, show that the generation approaching retirement age present a more deteriorated perceived health status compared to that of previous generations. Although some objective health indicators show favourable trends (reduction, albeit slowing, in mortality and incidence of several cardiovascular diseases), others appear less favourable (disability-free life expectancy, obesity, mental health) and, in particular, those relating to perceived health status and quality of life. These later must be regarded as complementary to objective health indicators for the assessment of population health. The question of how these trends will be reflected in terms of the burden of disability at later ages remains open and should encourage increased monitoring of this population in future years.

The results of this study should also serve as both a warning and encouragement to continue implementing health promotion and preventive actions in the field of healthy ageing in order to support successful ageing among the generations approaching retirement age. Furthermore, these results support the implementation of health promotion and preventive actions from middle age in the most vulnerable populations in order to reduce health inequalities and more specific actions relating to occupational health, to reduce the burden of mental health in particular.

## Supplementary Information

**Additional file 1: Supplementary file 1.** Wording of questions in the 2010 and 2017 Barometer surveys.

**Additional file 2: Supplementary file 2.** Characterics of people aged 55–65 in 2010 and 2017 according to socioeconomic determinants.

**Additional file 3: Supplementary file 3.** Change in perceived health between 2010 and 2017 according to socioeconomic determinants (relative risk ratios and 95% confidence interval), bivariate models, among those aged 55–65, by gender.

## Data Availability

The datasets used and analysed during the current study are not publicly available. Data are available on request from Sante publique France; any request must be addressed to Jean-Baptiste Richard (Jean-baptiste.richard@santepubliquefrance.fr). All data generated or analysed during this study are included in this published article and its supplementary information files.

## Abbreviations

MEHM: Minimum European Health Module
SRH: Self-reported health
GALI: Global Activity Limitation Indicator
WHO: World Health Organisation
ECHIM: European Community Health Indicators & Monitoring
RRR: Relative risk ratio
PR: Prevalence ratio
SF-36: The Short Form (SF-36) Health Survey
Promis-29: Patient Reported Outcome Measurement Information System
