# Supplementary material for: Alteration in perceived health status of those aged 55 to 65 between 2010 and 2017 in France: role of socioeconomic determinants
Source: BMC Public Health. 2021 Oct 7;21:1804. doi: 10.1186/s12889-021-11774-w (PMC8499534; doi:10.1186/s12889-021-11774-w)
Supplement: Supplementary file 1 — Additional file 1: Supplementary file 1. Wording of questions in the 2010 and 2017 Barometer surveys. [file 12889_2021_11774_MOESM1_ESM.docx]

**Supplementary file 1. Wording of questions in the 2010 and 2017 Barometer surveys.**

*Self-rated health:*

In 2017: “How is your health in general? Very good / Good / Fair / Bad / Very bad”

In 2010: “Overall, would you say that your health is: excellent / very good / good / average / bad”

*Chronic condition:*

In 2017: “Do you have any chronic or long-standing illnesses or health problems? Yes/No”

In 2010: “Do you have a chronic condition i.e. a long-standing illness (at least six months) that may require regular treatment (for example, diabetes, asthma, etc.)? Yes/No”

*Limitation:*

In 2017: “For at least the past six months, have you been limited in activities people usually do because of a health problem? Yes, severely limited / Yes, limited, but not severely / No, not limited at all”

In 2010: “For at least the past six months, have you been limited in activities people usually do because of a health problem or disability? No / Yes, but not severely / Yes, severely”
